# The Role of Ubiquitin in Regulating Stress Granule Dynamics

**DOI:** 10.3389/fphys.2022.910759

**Published:** 2022-05-25

**Authors:** Laura J. Krause, Maria G. Herrera, Konstanze F. Winklhofer

**Affiliations:** ^1^ Department of Molecular Cell Biology, Institute of Biochemistry and Pathobiochemistry, Ruhr University Bochum, Bochum, Germany; ^2^ RESOLV Cluster of Excellence, Ruhr University Bochum, Bochum, Germany

**Keywords:** FUS, G3BP, SUMO, TDP-43, ubiquitin, VCP/p97, LLPS

## Abstract

Stress granules (SGs) are dynamic, reversible biomolecular condensates, which assemble in the cytoplasm of eukaryotic cells under various stress conditions. Formation of SGs typically occurs upon stress-induced translational arrest and polysome disassembly. The increase in cytoplasmic mRNAs triggers the formation of a protein-RNA network that undergoes liquid-liquid phase separation when a critical interaction threshold has been reached. This adaptive stress response allows a transient shutdown of several cellular processes until the stress is removed. During the recovery from stress, SGs disassemble to re-establish cellular activities. Persistent stress and disease-related mutations in SG components favor the formation of aberrant SGs that are impaired in disassembly and prone to aggregation. Recently, posttranslational modifications of SG components have been identified as major regulators of SG dynamics. Here, we summarize new insights into the role of ubiquitination in affecting SG dynamics and clearance and discuss implications for neurodegenerative diseases linked to aberrant SG formation.

## Introduction

The evolution of eukaryotic cells involved a marked increase in organizational complexity, facilitated by intracellular compartmentalization (Gabaldón and Pittis, 2015; Rout and Field, 2017; Antifeeva et al., 2022). This was accomplished by the formation of cellular organelles by an endomembrane system and the acquisition of mitochondria, which developed from an alpha-proteobacterium engulfed by a eukaryotic progenitor. Spatio-temporal regulation of cellular processes through organelles increases the efficiency of cellular activities by concentrating components in the correct subcellular location and by avoiding interference with other cellular processes. A major barrier between the cytoplasm and different organelles are lipid membranes due to their capacity to exclude the diffusion of hydrophilic molecules, which therefore require uptake by selective transport mechanisms (Gabaldón and Pittis, 2015; Honigmann and Pralle, 2016). In recent years, another type of cellular compartmentalization has been studied intensively that is characterized by the transient formation of membrane-less organelles. These structures are formed by the condensation of proteins and nucleic acids and are also called biomolecular condensates (Banani et al., 2017; Wheeler and Hyman, 2018; Alberti et al., 2019; Gomes and Shorter, 2019; Dignon et al., 2020). A characteristic feature of biomolecular condensates is their highly dynamic behavior, allowing cells to promptly respond to cellular stress or changes in the metabolic demand and to process local information in the context of cellular signaling. Biomolecular condensates are formed in the nucleus, such as nucleoli, Cajal bodies, nuclear speckles, and paraspeckles, and also occur in the cytoplasm, with SGs, P-bodies, and germ granules being the most studied examples.

SGs are assembled in response to various stress conditions, including oxidative, osmotic and heat stress, UV radiation, and viral infections (Protter and Parker, 2016; Riggs et al., 2020; Tauber et al., 2020). Their size ranges from 200 to 1,000 nm and they are mainly composed of proteins and non-translating messenger ribonucleic acids (mRNAs). The formation of SGs occurs within an adaptive stress response program to protect cells under acute stress by a reversible shutdown of several cellular activities and translational reprogramming. In this process, house-keeping mRNAs are stalled and recruited to SGs, whereas mRNAs coding for stress-responsive proteins, such as heat shock proteins (HSPs), are excluded from SGs, facilitating the expression of proteins required for coping with cellular stress (Franzmann and Alberti, 2019). In addition, SGs can sequester various molecules unrelated to RNA metabolism, suggesting a role in regulating cellular signaling pathways, such as anti-viral, tumor necrosis factor (TNF), and mechanistic target of rapamycin (mTOR) signaling (Kim et al., 2005; Takahara and Maeda, 2012; Protter and Parker, 2016; Eiermann et al., 2020; Prentzell et al., 2021). From a biophysical perspective, the formation of SGs is governed by liquid-liquid phase separation (LLPS). This is a thermodynamically driven, reversible process that involves de-mixing of two liquid phases through protein-protein, protein-RNA, and RNA-RNA interactions (Shin and Brangwynne, 2017; Mathieu et al., 2020). *In vitro*, LLPS can be influenced by various physicochemical parameters, such as temperature, pH, osmolarity, ionic strength, and macromolecular crowding (Molliex et al., 2015; Alberti et al., 2019; Cinar et al., 2019; Dignon et al., 2020). However, these parameters are difficult to modify in a cell-autonomous manner. Hence, cells employ posttranslational modifications (PTMs) to rapidly and reversibly regulate LLPS and the assembly and disassembly of biomolecular condensates by strengthening or weakening multivalent interactions (Rhoads et al., 2018; Wheeler and Hyman, 2018; Hofweber and Dormann, 2019). In particular, phosphorylation, acetylation, methylation, poly-ADP-ribosylation, ubiquitination and modification by the ubiquitin-like proteins small ubiquitin-related modifier (SUMO) and neural precursor cell expressed developmentally downregulated protein (NEDD) have been implicated in the assembly and disassembly of SGs (Tourrière et al., 2003; Jayabalan et al., 2016; Jongjitwimol et al., 2016; Tsai et al., 2016; Reineke et al., 2017; Duan et al., 2019; Gal et al., 2019; Saito et al., 2019; Huang et al., 2020; Keiten-Schmitz et al., 2020). PTMs affect the physicochemical characteristics of the modified proteins, for example their charge, hydrophobicity, and conformation, which can influence their LLPS behavior by affecting their valency and thus their interaction properties (Farina et al., 2021; Sternburg et al., 2022).

**FIGURE 1:**
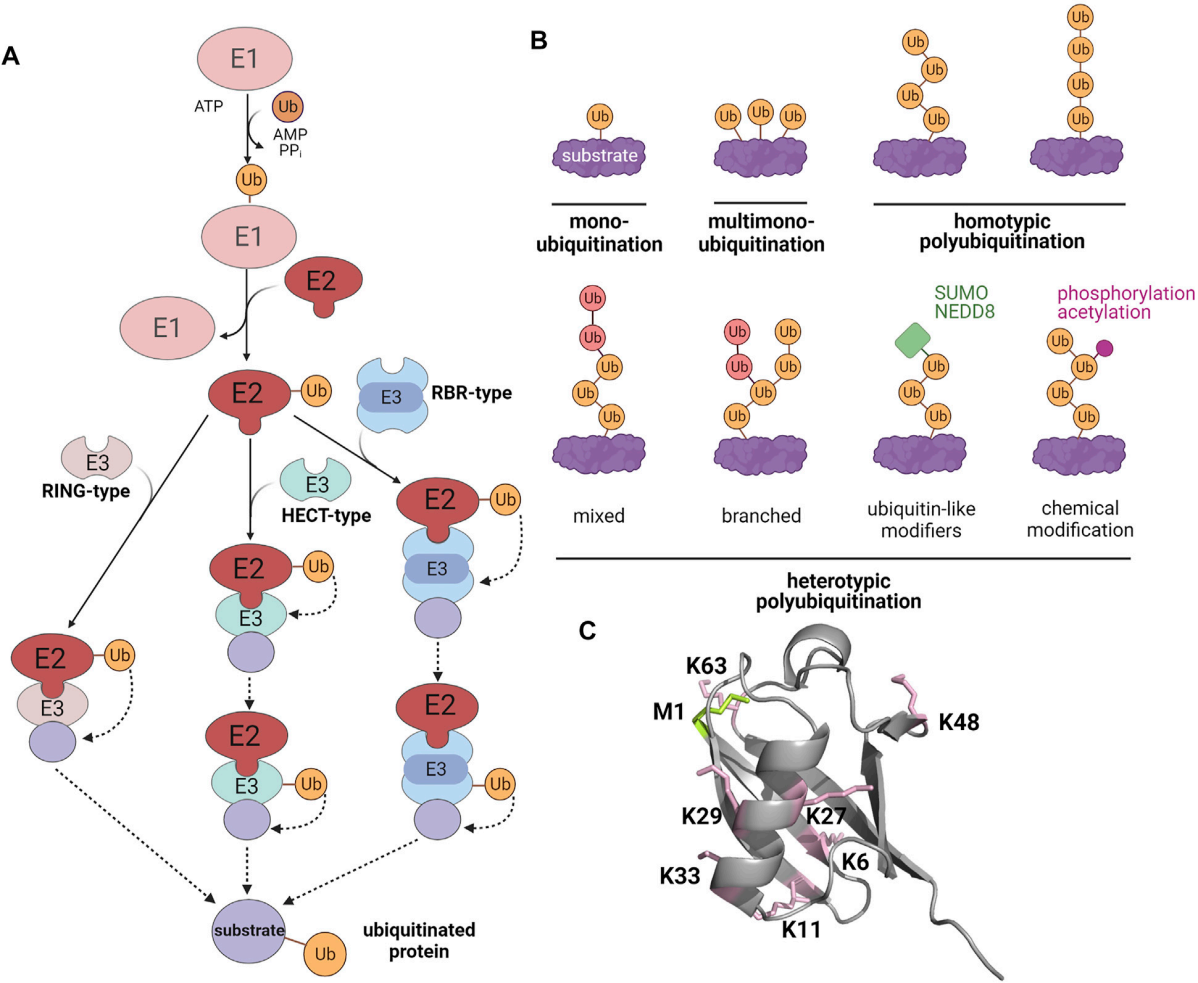
The modification of substrate proteins by ubiquitin. **(A)** Enzymatic cascade of ubiquitination. E1, ubiquitin-activating enzyme; E2, ubiquitin-conjugating enzyme; E3, ubiquitin ligase. **(B)** Different types of ubiquitination **(C)** X-ray crystallographic structure of ubiquitin (PDB: 1UBQ). Lysines are highlighted in pink and the methionine at the N-terminus is shown in green. Created with BioRender.com.

In the regulation of manifold cellular processes, including protein quality control and signaling, ubiquitination has moved to center stage. The covalent modification of substrates with ubiquitin requires a tightly controlled three-step enzymatic cascade (Berndsen and Wolberger, 2014; Swatek and Komander, 2016; Oh et al., 2018). Firstly, an E1 ubiquitin-activating enzyme uses ATP to adenylate the C-terminus of ubiquitin, allowing thioester formation between ubiquitin and the E1 active site cysteine. Then, ubiquitin is transferred to an E2 ubiquitin-conjugating enzyme by trans-thioesterification. Finally, ubiquitin is conjugated to a lysine (K) residue of the substrate protein by specific E3 ubiquitin ligases. E3 ligases belonging to the really interesting new gene (RING)/U-box families facilitate the direct transfer of ubiquitin from the E2 enzyme to the substrate. Homologous to E6-AP carboxyl terminus (HECT) E3 ligases form a thioester intermediate with ubiquitin by an active site cysteine, from which ubiquitin is transferred onto the substrate to form an isopeptide bond. RING-between-RING (RBR) ligases use a RING/HECT hybrid mechanism. They bind to their cognate E2 enzyme with their RING1 domain and then ligate ubiquitin to the catalytic cysteine within their RING2 domain by forming a transient thioester (Figure 1). Ubiquitination is counteracted by deubiquitinases (DUBs), which show selectivity for either the ubiquitin chain topology or the ubiquitinated substrate (Mevissen and Komander, 2017; Clague et al., 2019). A characteristic feature of ubiquitination is its high versatility based on variable numbers of ubiquitin moieties attached to a substrate (mono- or polyubiquitination), the type of ubiquitin linkage within polyubiquitin chains, and the formation of homo- or heterotypic polyubiquitin chains with mixed or branched chain architecture. The linkage between ubiquitin molecules occurs between the C-terminal glycine of the donor ubiquitin and one of seven lysine residues (K6, K11, K27, K29, K33, K48, K63) or the N-terminal methionine (M1) of the acceptor ubiquitin. The complexity of the ubiquitin code is further increased by PTMs of ubiquitin itself, such as phosphorylation, acetylation, SUMOylation, and NEDDylation (Figure 1) (Swatek and Komander, 2016; Yau and Rape, 2016; Oh et al., 2018). Ubiquitination plays a major role in the regulation of cellular proteostasis and signaling, suggesting that it may also affect SG dynamics. In support of this notion, several proteins of the ubiquitination machinery are SG components (Jain et al., 2016; Markmiller et al., 2018; Marmor-Kollet et al., 2020). Moreover, mutations of some of these SG components are linked to neurodegenerative diseases that are characterized by defective SG dynamics and the accumulation of polyubiquitinated protein aggregates (Shin and Brangwynne, 2017; Alberti and Dormann, 2019; Nedelsky and Taylor, 2019; Mathieu et al., 2020; Zbinden et al., 2020; Lei et al., 2021). Pathological mutations in SG-related RBPs can also influence the SG proteome. Markmiller et al. (2018) observed that the SG composition, dynamics, and localization are different in motor neurons with the Amyotrophic Lateral Sclerosis (ALS)-linked mutations in chromosome nine open reading frame 72 (C9orf72) or heterogeneous nuclear ribonucleoprotein A2/B1 (hnRNPA2/B1).

Several lines of evidence indicated that an impaired disassembly of SGs promotes their conversion from a liquid to a gel- or solid-like state, favoring the formation of pathological aggregates (Shin and Brangwynne, 2017; Wolozin and Ivanov, 2019; Zhang et al., 2019). Aggregation of SG-associated proteins occurs in several neurodegenerative disorders, such as ALS and Frontotemporal Dementia (FTD). In these diseases, pathogenic mutations have been identified in the RNA-binding proteins (RBPs) TAR DNA-binding protein 43 (TDP-43), fused in sarcoma (FUS), T-cell internal antigen-1 (TIA-1), hnRNPA1/A2, matrin-3 (MATR3), and in the ubiquitin-binding proteins ubiquilin 2 (UBQLN2), valosin-containing protein (VCP/p97), optineurin (OPTN), and sequestosome-1 (SQSTM1/p62) (Patel et al., 2015; Taylor, 2015; Mackenzie et al., 2017). Interestingly, aggregates formed by these proteins stain positive for ubiquitin in brain sections from affected patients, suggesting a role of ubiquitination (Galves et al., 2019). In this review, we summarize our current knowledge on ubiquitin and ubiquitin-like modifiers as regulators of SG dynamics.

## STRESS GRANULES Are Formed by Liquid-Liquid Phase Separation

SGs are classified as ribonucleoprotein (RNP) granules, in which homotypic interactions, such as protein-protein and RNA-RNA interactions, as well as heterotypic protein-RNA interactions occur (Mittag and Parker, 2018; Van Treeck et al., 2018; Van Treeck and Parker, 2018). These condensates show a dynamic behavior, allowing shuttling of proteins and RNA between SGs and the cytoplasm. Under thermodynamically favorable conditions, biomolecular condensates are formed by exchanging macromolecule-water interactions to macromolecule-macromolecule and water-water interactions (Alberti et al., 2019; Ribeiro et al., 2019; Mathieu et al., 2020).

Specific regions of proteins and RNAs can establish multivalent interactions similarly to associative polymers (Brangwynne et al., 2015). In this way, they form reversible, non-covalent physical cross-links and thereby promote LLPS. According to the concept of “stickers and spacers,” short linear motifs (SLiMs) of 1–10 residues function as stickers that promote interactions with other biomolecules (Choi et al., 2020). Most of the stickers have arginine-glycine-rich (RGG/RG) or serine/arginine-rich motifs (Chong et al., 2018). Small polar residues and a small fraction of aromatic and charged residues are also typically present in these motifs (Martin et al., 2020; Borcherds et al., 2021). The “spacers” are mainly disordered regions that connect the stickers. Their number and length influence the formation of biomolecular condensates. Proteins undergoing LLPS typically harbor RNA recognition motifs (RRMs), intrinsically disordered regions (IDR), or regions with little diversity in their residue composition, called low complexity domains (LCDs) (Molliex et al., 2015; Martin and Mittag, 2018; Wheeler and Hyman, 2018; Franzmann and Alberti, 2019; Youn et al., 2019). These proteins are further classified in “scaffold” proteins required for the assembly of biomolecular condensates and “clients,” which are non-essential (Ishov et al., 1999; Banani et al., 2016, 2017). Based on super-resolution microscopy and proteomic studies, it has been proposed that SGs are composed of a stable core and a more dynamic shell (Jain et al., 2016; Wheeler et al., 2016; Markmiller et al., 2018; Youn et al., 2018).

Molecular forces that mainly govern LLPS are weak multivalent interactions, such as electrostatic, π-π, cation-π, and hydrophobic interactions (Li et al., 2012; Sherrill, 2013; Nott et al., 2015; Kim et al., 2016; Lin et al., 2016; Pak et al., 2016; Pierce et al., 2016; Kang et al., 2019; Das et al., 2020). These interactions are influenced by structural characteristics and the concentration of the macromolecules involved as well as by environmental conditions, such as temperature, pH, the presence and concentration of salts, co-solutes and crowding agents (Molliex et al., 2015; Nott et al., 2015; Patel et al., 2015; Alberti et al., 2019; Dignon et al., 2020). Both the chemical milieu and PTMs can induce conformational alterations of proteins, which influence their intra- and intermolecular interactions (Csizmok and Forman-Kay, 2018; Mittag and Parker, 2018; Van Treeck and Parker, 2018; Youn et al., 2019; Yang et al., 2020).

Defective disassembly of SGs and their maturation into aggregates is favored by persistent stress and mutations in RBPs genes, such as TDP-43, FUS, and hnRNPA1, linked to neurodegenerative diseases. In support of this notion, SG markers colocalize with protein aggregates in affected brain regions (Liu-Yesucevitz et al., 2010; Dormann and Haass, 2011; Li et al., 2013). Disease-associated mutations as well as PTMs of RBPs affect the conformational plasticity of SG components and their interactive profile, thereby influencing their transition into aggregates (Bentmann et al., 2013; Patel et al., 2015; Ratti and Buratti, 2016; Dobra et al., 2018; McGurk et al., 2018).

## Stress Granules Are Phase-Separated Condensates That Quarantine RNAs and Proteins Under Cellular Stress

**FIGURE 2:**
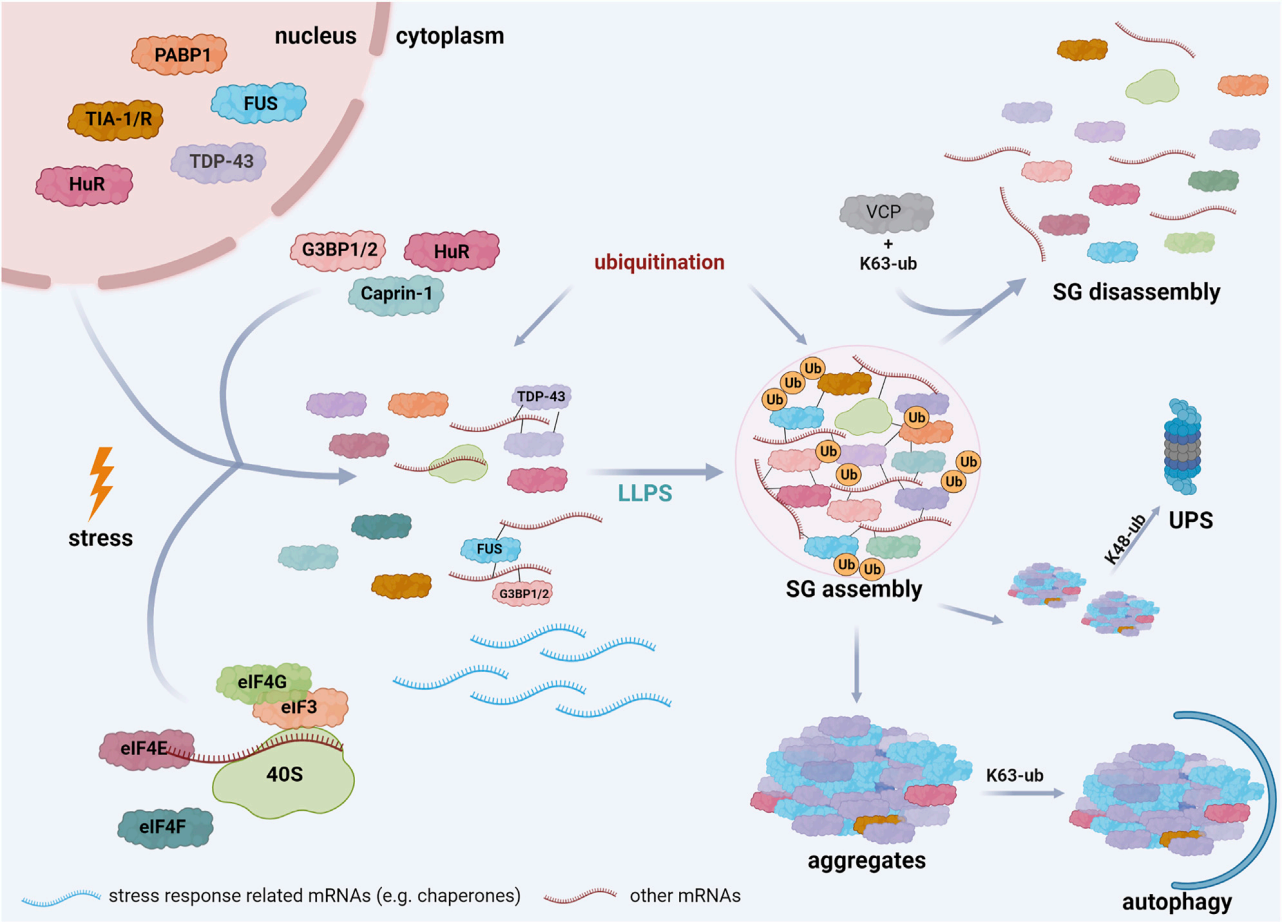
Schematic representation of stress granule assembly in the cell. Cells exposed to several stress conditions respond by inhibiting translation initiation of mRNAs that are not implicated in stress responses. These mRNAs interact with specific RBPs, such as TDP-43, FUS, G3BP, and undergo LLPS when a critical threshold of interactions is reached, resulting in SG formation. Both SG assembly and disassembly are regulated by PTMs, including ubiquitination. SG disassembly is promoted by K63-linked ubiquitination of G3BP and the interaction of these ubiquitin chains with VCP. Mutations in SG components, prolonged stress, and PTM dysregulation can induce the transition of SGs into pathological aggregates. The elimination of these aggregates is regulated by ubiquitination; K48-linked ubiquitin chains are well established signals for degradation by the ubiquitin-proteasome system (UPS), whereas K63-linked ubiquitination is rather implicated in autophagosomal degradation. Created with BioRender.com.

SGs are mostly composed of three types of proteins: translation factors, including ribosomal subunits, RBPs, and other proteins, such as several enzymes. For more details, we refer to databases of SG proteins (https://msgp.pt; http://rnagranuledb.lunenfeld.ca; Nunes et al., 2019; Youn et al., 2019). Regarding the first class of proteins, translation factors like eukaryotic translation initiation factor 3 (eIF3), eIF4E, eIF4F, eIF4G, and the ribosomal 40 S subunit have been identified as SG components (Kedersha and Anderson, 2002; Kedersha et al., 2002). RBPs detected in SGs include Ras GTPase-activating protein (SH3 domain)-binding proteins (G3BPs) (Tourrière et al., 2003; Markmiller et al., 2018), TIA-1 (Markmiller et al., 2018), TIA-1-related protein (TIAR), polyadenylated [poly(A)^+^] mRNA binding protein I (PABP-I) (Kedersha et al., 1999, Kedersha et al., 2002; Kedersha and Anderson, 2002), survival motor neuron protein (SMN) (Hua and Zhou, 2004), Staufen (Thomas et al., 2005), ataxin-2, Caprin-1, fragile X mental retardation protein 1 (FMRP1), DEAD box protein 1 (DDX1) (Markmiller et al., 2018), TDP-43 (Aulas et al., 2012), FUS (Wolozin, 2012; Li et al., 2013) and Human antigen R (HuR) (Bley et al., 2015) (Figure 2). ATPases, such as VCP/p97 (Wang et al., 2016), the co-chaperone HspBP1 (Mahboubi et al., 2020) and kinases, including Unc-51-like autophagy activating kinase 1 and 2 (ULK1/2) (Wang et al., 2019) have also been associated to SGs. The identification of ubiquitin E3 ligases, like Makorin-1, RING finger protein 241 (RNF214) and tripartite motif protein family members (TRIM21, TRIM25, TRIM56) (Cassar et al., 2015; Jain et al., 2016; Marmor-Kollet et al., 2020) and DUBs, such as ubiquitin-specific-processing proteases (USP5, USP10, USP11) and the OTU (ovarian tumor)-domain containing protein 4 (OTUD4) (Jain et al., 2016; Markmiller et al., 2018) strongly support the notion that ubiquitination controls SG-related processes.

The assembly of SGs is initiated by at least two events: the phosphorylation of eIF2α or the inactivation of eIF4A. eIF2α is phosphorylated by several kinases, including protein kinase RNA-dependent kinase (PKR), PKR-like endoplasmic reticulum kinase (PERK), heme-regulated initiation factor 2α kinase (HRI), and general control non-derepressible protein 2 (GCN2), which are activated in different stress conditions, such as heat, oxidative, and UV stress (Lu et al., 2001; Deng et al., 2002; McEwen et al., 2005). Phosphorylation of eIF2α inhibits translation initiation by depleting the eIF2/tRNA_i_^Met^/GTP ternary complex, resulting in the formation of translationally stalled non-canonical 48 S complexes that are not able to recruit the 60 S ribosomal subunit (Kedersha et al., 1999, 2002; McCormick and Khaperskyy, 2017). Alternatively, pharmacological inhibition of the helicase eIF4A by hippuristanol or pateamine A blocks its interaction with eIF4G, leading to translation inhibition and the formation of SGs independent of eIF2α phosphorylation (Bordeleau et al., 2005; Low et al., 2005; McCormick and Khaperskyy, 2017; Hofmann et al., 2021). Both mechanisms lead to an increase in the cellular concentration of mRNAs, which interact with various RNA-binding proteins. G3BPs play a critical role in SG formation, since binding of RNAs to G3BPs induces a conformational transition of G3BPs that allows the formation of a protein-RNA network driving LLPS (see Section 5.4); (Guillén-Boixet et al., 2020; Sanders et al., 2020; Yang et al., 2020) (Figure 2). As a transient adaptive stress response, SGs disassemble when the stress is removed, allowing recycling of mRNAs and sequestered proteins and re-establishment of cellular activities.

## The Role of Ubiquitin and Ubiquitin-Like Proteins in STRESS GRANULE Assembly, Disassembly, and Clearance

### Ubiquitin and SGs

Ubiquitination can in principle affect all steps of SG formation, dynamics and elimination. Notably, the composition and dynamics of SGs are dependent on the specific stress stimulus and the cell type, in which they are formed (Aulas et al., 2017; Markmiller et al., 2018; Yang et al., 2020). These variables most likely also determine the effect of ubiquitination on SG dynamics and clearance. Moreover, different ubiquitin chain architectures based on specific inter-ubiquitin linkages, the formation of heterotypic ubiquitin chains and posttranslational modifications of ubiquitin itself increase the complexity of possible regulatory roles of ubiquitin. Recent publications substantiated an important role of K63-linked ubiquitination in the disassembly of heat-stress-induced SGs and thus in the recovery from stress (Gwon et al., 2021; Maxwell et al., 2021). However, also other aspects seem to be affected by ubiquitination. Kwon et al. (2007) observed that SGs are ubiquitinated and recruit the histone deacetylase 6 (HDAC6) in a ubiquitin-dependent manner. The authors of this study reported that the deacetylase activity of HDAC6 is required for SG formation under different stress conditions by mediating the motor protein-driven movement of SG proteins along microtubules. In a screen performed in *Saccharomyces cerevisiae* to identify genes that affect SG assembly, deletion of the histone E3 ligase 2 (Hel2) was found to increase SG formation (Buchan et al., 2013). Another study reported that inhibition of the E3 ubiquitin ligase anaphase promoting complex (APC) and its regulatory subunit Cadherin-1 (Cdh1) in primary cortical neurons increases SG formation in a manner that is dependent upon its substrate FMRP (Valdez-Sinon et al., 2020). These data suggested that ubiquitination of some SG components interferes with their efficient incorporation into SGs. In a study comparing different stress conditions in HeLa cells, K48- and K63-linked ubiquitin chains were detected at SGs induced by heat stress, osmotic stress, and arsenite treatment. Inhibition of the E1 ubiquitin-activating enzyme, VCP/p97, or the proteasome impaired the clearance of arsenite- and heat-stress-induced SGs (Tolay and Buchberger, 2021). E3 ubiquitin ligases have also been linked to virus-induced SG formation. Kuniyoshi et al. (2014) found that the RNA-binding E3 ubiquitin ligase mex-3 homolog C (MEX3C), which is the mammalian homolog of *Caenorhabditis elegans* RNA-binding protein muscle excess 3 (MEX-3), colocalizes with the viral SG component retinoic acid-inducible gene I (RIG-I), a cytoplasmic receptor for viral RNAs that induces antiviral innate immune responses, such as expression of type I interferons (IFN). Increased expression of MEX3C induced K63-linked ubiquitination of RIG-I and activation of the IFN-ß promoter, thereby enhancing the type I IFN antiviral response (Kuniyoshi et al., 2014). In a recent publication on the ubiquitination landscape upon heat stress, ubiquitination of SG components, including FUS, TDP-43, and some DDX family proteins was observed (see Section 5.4) (Maxwell et al., 2021). Interestingly, in this study heat stress but not arsenite treatment induced the ubiquitination of SG components, which was important for SG disassembly and recovery from stress by a VCP/p97-dependent process (Maxwell et al., 2021). This is in line with the observation that ubiquitination is dispensable for the dynamics of arsenite-induced SGs (Markmiller et al., 2019). Another study provided evidence for a role of K63-linked ubiquitination of G3BP1 in the disassembly of SGs formed under heat stress. Ubiquitinated G3BP1 interacts with the ER-associated protein FAS-associated factor 2 (FAF2) that engages the segregase VCP/p97 to extract G3BP1 from SGs, resulting in SG disassembly (Gwon et al., 2021). The disassembly of arsenite-induced SGs was recently analyzed by proximity proteomics, revealing several proteins that are recruited during SG disassembly, called disassembly-engaged proteins (DEPs). These DEPs include SUMO-conjugating enzymes as well as proteins of the ubiquitin machinery, such as the E3 ligases HECT domain-containing protein 4 (HECTD4), male-specific lethal-2 (MSL2), RING finger protein 41 (RNF41), X-linked inhibitor of apoptosis protein (XIAP), the E2 enzyme UBE2G2, and the DUB USP25 (Marmor-Kollet et al., 2020).

The mechanisms implicated in SG clearance are dependent on the severity and duration of the initiating stress. Gwon et al. (2021) reported that SGs induced by a heat stress of 30 or 60 min can be disassembled, whereas SGs formed upon a prolonged heat stress of 90 min need to be cleared by autophagy. Ubiquitination of SG components not only influences SG dynamics but also affects their elimination. Persistent SGs occurring under prolonged stress or disease conditions, e.g., through pathogenic mutations of SG components, can be cleared by autophagy in a process called granulophagy that requires VCP/p97 (see below) (Buchan et al., 2013).

### SUMO, NEDD8 and Stress Granules

In addition to ubiquitin, the ubiquitin-like modifiers SUMO and NEDD8 can regulate SG dynamics. SUMOylation and NEDDylation also require a set of three enzymes (E1, E2, and E3) for the covalent modification of substrate proteins (Flotho and Melchior, 2013; Enchev et al., 2015). SUMOylation of eIF4A has been implicated in SG formation after arsenite treatment and ionization radiation (Jongjitwimol et al., 2016). SUMOylation of eIF4A correlated with its recruitment to SGs, whereas a SUMOylation-deficient eIF4A2 mutant decreased the volume of SGs. A role for SUMO in SG disassembly and formation was provided by Marmor-Kollet et al. (2020) who identified proteins of the SUMOylation machinery as DEPs, including SUMO-activating enzyme subunit 1 (SAE1), SUMO-conjugating enzyme ubiquitin carrier protein 9 (UBC9), E3 SUMO-protein ligase Ran-binding protein 2 (RanBP2), and the E3 ubiquitin/SUMO-protein ligase Topors. In support of this notion, inhibition of SUMOylation or silencing of SAE1 or UBC9 interfered with SG dynamics (Marmor-Kollet et al., 2020). Overexpression of a GFP fusion construct with 50 proline-arginine dipeptide repeats, linked to the C9orf72 subtype of ALS, impaired conjugation of SUMO2/3 to SGs, DEP recruitment, and SG disassembly in U2OS cells. Keiten-Schmitz et al. (2020) provided evidence for a role of the nuclear SUMO-targeted ubiquitin ligase (StUbL) pathway in regulating cytoplasmic SG dynamics. Proteotoxic stress inactivates SUMO isopeptidases, resulting in an increase in SUMOylation and activation of the SUMO-dependent nuclear E3 ubiquitin ligase RNF4. SUMO-primed RNF4-dependent ubiquitination of SG-related RBPs has been shown to promote SG disassembly in the cytoplasm by a yet unknown crosstalk between the nuclear and cytoplasmic quality control. Proteasomal degradation of ubiquitinated RBPs in the nucleus or prevention of their aggregation might be involved in this process (Keiten-Schmitz et al., 2020).

The impact of NEDDylation on SG dynamics is a controversial issue. Markmiller et al. (2019) reported that pharmacological inhibition of the NEDD8-activating enzyme does not affect arsenite-induced SG dynamics. In contrast, Jayabalan et al. (2016) observed that silencing NEDD8 or the NEDD8-conjugating enzyme UBC12 impairs arsenite-induced SG assembly. In this study, translationally stalled ribosomal fractions were analyzed for NEDDylated proteins by mass spectrometry and conjugation of NEDD8 to the SG component serine/arginine-rich splicing factor 3 (SRSF3) was shown to promote SG assembly. One possible explanation for these discrepant findings could be cell type-specific effects. Whereas Markmiller et al. (2018), Markmiller et al. (2019) used HEK293T and HeLa cells, Jayabalan et al. (2016) performed their study in U2OS cells.

## Specific STRESS GRANULE Proteins and Their Regulation by Ubiquitin

Defective dynamics of SGs, their persistence and transition into aggregates have been linked to neurodegenerative diseases, such as ALS and FTD. Notably, mutations in several ubiquitin-binding proteins, such as p62/SQSTM1, UBQLN2, OPTN, and VCP/p97 cause neurodegeneration, underpinning the role of ubiquitin in proteostasis regulation (Pohl and Dikic, 2019; Lei et al., 2021). Both p62 and UBQLN2 form phase-separated condensates under different stress conditions that are regulated by ubiquitin. The proteasomal shuttle factor UBQLN2 binds to SGs and undergoes LLPS that is inhibited by mono- and polyubiquitin. This suggests that ubiquitination serves as a switch between UBQLN2 recruitment to SGs and UBQLN2-dependent shuttling of ubiquitinated SG components (Dao et al., 2018). Phase separation of the ubiquitin-binding and autophagy adaptor protein p62 is dependent on its oligomerization and binding to polyubiquitinated cargo, which promotes condensation of polyubiquitinated proteins and tethering to the autophagosomal membrane for selective autophagy (Sun et al., 2018; Zaffagnini et al., 2018; Turco et al., 2019, 2021). In the following, we summarize the current knowledge on the disease-related proteins TDP-43, FUS and VCP/p97 and the SG scaffolds G3BPs and their regulation by ubiquitin.

### TDP-43

Human TDP-43 is an RNA-binding protein of 414 amino acids that is causally linked to ALS and FTD. In 2006, TDP-43-positive inclusions were first described in patients’ brains and TDP-43 was found to be hyper-phosphorylated, ubiquitinated, and proteolytically processed (Neumann et al., 2006, 2009a; Hasegawa et al., 2008). Subsequently, pathogenic mutations in the *TARDBP* gene encoding TDP-43 were identified in familial ALS and more rarely in FTD (Gitcho et al., 2008; Rutherford et al., 2008; Sreedharan et al., 2008). TDP-43 consists of four domains. The highly conserved N-terminal domain (NTD) comprises residues with a well-defined fold (Qin et al., 2014). The correct folding of this region, especially of the first ten residues, is crucial for the splicing activity of TDP-43 and its homodimerization into solenoid-like structures (Ayala et al., 2005; Afroz et al., 2017). The NTD is followed by two RRMs, RRM1 and RRM2, with two β-α-β folds that bind mostly UG/TG sequences (Maris et al., 2005). The NTD and RRM domains contain several caspase cleavage sites, generating C-terminal fragments of TDP-43 (Dormann et al., 2009; Li et al., 2015). The glycine-rich C-terminal region is less conserved and harbors most of the pathogenic mutations (Pesiridis et al., 2009; Dormann and Haass, 2011; Rademakers et al., 2012). This region, termed Prion-like domain (PrLD), is intrinsically disordered and promotes LLPS and aggregation of TDP-43 (Conicella et al., 2016; Nonaka and Hasegawa, 2018, Nonaka and Hasegawa, 2020; Loughlin and Wilce, 2019).

TDP-43 contains a bipartite nuclear localization and a nuclear export signal. It is mostly localized in the nucleus, where it regulates RNA metabolism, gene expression, and the DNA damage response (Buratti et al., 2004; Ratti and Buratti, 2016; Buratti, 2018; Birsa et al., 2020). Under several stress conditions, TDP-43 translocates to the cytoplasm, where it is recruited to SGs (Colombrita et al., 2009; Freibaum et al., 2010; Liu-Yesucevitz et al., 2010; Prpar Mihevc et al., 2017; Lee et al., 2021). Pathogenic TDP-43 mutants have been described to increase the size of SGs and to impair their disassembly, favoring their transition into aggregates (Dewey et al., 2011; Dobra et al., 2018).

The presence of ubiquitin in TDP-43 inclusions is a characteristic feature of TDP-43 pathologies. The most abundant form of TDP-43 in ALS and FTD inclusions are C-terminal fragments of about 25 kDa (Igaz et al., 2008). The ubiquitin-binding protein p62 is also found at TDP-43 inclusions, providing a link to protein quality control and degradation (Tanji et al., 2012). In cellular models, stress conditions associated with the formation of SGs, such as heat stress, osmotic stress, or arsenite treatment, induced ubiquitination, insolubility and reduced splicing activity of TDP-43 (Hans et al., 2020). Keiten-Schmitz et al. (2020) identified TDP-43 as a target of the SUMO-dependent nuclear E3 ubiquitin ligase RNF4. Silencing of RNF4 reduced heat stress-induced TDP-43 ubiquitination and impaired the disassembly of cytoplasmic SGs, suggesting a link between nuclear and cytoplasmic protein quality control pathways (see above). The E3 ubiquitin ligase Parkin has been reported to ubiquitinate TDP-43 and to facilitate its cytoplasmic accumulation in a multiprotein complex containing HDAC6 (Hebron et al., 2013). Moreover, TDP-43 was shown to regulate Parkin expression, resulting in a decreased abundance of Parkin in motor neurons from ALS patients with TDP-43 pathology, which may contribute to the mitochondrial defects observed in TDP-43 pathologies (Polymenidou et al., 2011; Lagier-Tourenne et al., 2012; Gaweda-Walerych et al., 2021). The E2 ubiquitin conjugating enzymes UBE2E and the ubiquitin isopeptidase Y (UBPY) were identified as TDP-43-interacting proteins that regulate the ubiquitination of wildtype TDP-43, TDP-43 mutants and C-terminal fragments (Hans et al., 2014). A study of ubiquitin homeostasis in cellular models of ALS revealed that aggregates formed upon the overexpression of GFP-tagged TDP-43 or FUS stained positive for K48- and K63-linked ubiquitin chains, in line with a role for proteasomal and autophagosomal degradation of these proteins (Urushitani et al., 2009; van Eersel et al., 2011; Scotter et al., 2014; Farrawell et al., 2020). These ubiquitin topologies were also identified in a proteomic analysis of a cellular model overexpressing TDP-43 and several ubiquitination sites within TDP-43 have been identified (Seyfried et al., 2010; Kim et al., 2011; Wagner et al., 2011; Dammer et al., 2012; Buratti, 2018; Hans et al., 2018; Lee et al., 2018). Overexpression of the TDP-43 mutant M337V in the motor cortex of monkeys resulted in the cytoplasmic aggregation of TDP-43 C-terminal fragments that were predominantly modified by K63-linked ubiquitin (Yin et al., 2021). Interestingly, van Well et al. (2019) observed colocalization of M1-linked ubiquitin chains with TDP-43 aggregates in cellular models and provided evidence for ubiquitination of TDP-43 by LUBAC, the linear ubiquitin chain assembly complex. The M1 ubiquitin-specific E3 ligase HOIL-1-interacting protein (HOIP, also known as RNF31), which is the catalytically active component of LUBAC, reduced the fraction of cells with TDP-43 aggregates in a proteasome- and VCP/p97-dependent manner, indicating a role for M1-linked ubiquitination in protein quality control (van Well et al., 2019). Supporting a relevance of these findings *in vivo*, M1-linked ubiquitin and HOIP were detected at neuronal TDP-43 inclusions in sporadic ALS patients (Nakayama et al., 2020).

### FUS

FUS is a RNA-binding protein of 526 amino acids, which is primarily localized in the nucleus and involved in various aspects of RNA metabolism. It is composed of an N-terminal LCD rich in glutamine/glycine/serine/tyrosine residues followed by three arginine/glycine-rich domains interspersed by an RRM and a zinc finger domain. FUS has a C-terminal nuclear localization signal that is affected by several pathogenic mutations, causing mislocalization and aggregation of FUS in the cytoplasm (Bosco et al., 2010; Dormann et al., 2010; Kino et al., 2011). FUS has various functions in transcription, splicing, translation, and DNA damage repair (Chen et al., 2019; Birsa et al., 2020; Sternburg et al., 2022). In subtypes of ALS and FTD, FUS aggregates in specific brain regions and mutations in the *FUS* gene have been linked to familial ALS (Neumann et al., 2009b; Kwiatkowski et al., 2009; Vance et al., 2009). FUS can undergo LLPS and pathogenic FUS mutations have been shown to enhance the phase transition of stress-induced FUS condensates, influencing their dynamics and promoting their transition into aggregates (Bosco et al., 2010; Patel et al., 2015; Wang et al., 2018; Sternburg et al., 2022). An important PTM of FUS is arginine methylation, which regulates its nuclear import by the nuclear import receptor transportin-1 (Hofweber et al., 2018). Hypomethylation of FUS is a characteristic feature of FTD in patients, which is supposed to increase LLPS of FUS and its maturation into aggregates (Dormann et al., 2012; Suárez-Calvet et al., 2016; Hofweber et al., 2018; Qamar et al., 2018).

The colocalization of FUS with ubiquitin in pathological aggregates suggests that FUS is ubiquitinated, which is supported by proteomic approaches (Neumann et al., 2009b; Mertins et al., 2013; Akimov et al., 2018; Farrawell et al., 2020; Sternburg et al., 2022). FUS aggregates formed by the overexpression of GFP-tagged mutant FUS in a cellular model accumulated K48- and K63-linked ubiquitin chains, which reduced the free ubiquitin pool and disrupted ubiquitin homeostasis (Farrawell et al., 2020). Similarly to TDP-43, FUS was shown to be modified by the SUMO-dependent nuclear E3 ubiquitin ligase RNF4 upon heat stress and the inhibition of this modification promoted the recruitment of mutant FUS to SGs (Keiten-Schmitz et al., 2020).

### VCP/p97

The human AAA+ (ATP-ase associated with various cellular activities) protein VCP/p97 is a 806 residue protein that is highly conserved from yeast to humans (Barthelme and Sauer, 2016). It is a key enzyme of the protein quality control network that employs ATP to unfold and segregate ubiquitinated proteins from cellular membranes, oligomeric assemblies, or protein aggregates (van den Boom and Meyer, 2018; Zhang and Mao, 2020). VCP/p97 is a ring-shaped hexamer, in which each monomer is composed of a globular NTD, two ATPase domains (D1 and D2), and a disordered tail. The NTD interacts with several ubiquitin-binding cofactors, such as the Ufd1-Npl4 complex, which function as ubiquitin adaptors and recruit ubiquitinated client proteins to VCP/p97 (Dai and Li, 2001; Buchberger et al., 2015). The D1 and D2 regions interact with each other, producing two stacked hexameric rings. VCP/p97 has a plethora of functions at different cellular locations, for example at the ER membrane, at the outer mitochondrial membrane, and in the nucleus. Based on its ability to extract ubiquitinated proteins from membranes and protein assemblies, it not only plays an important role in protein quality control by delivering client proteins to the proteasome, but also regulates ubiquitin-dependent signaling and chromatin-associated functions. Moreover, several studies provided evidence that VCP/p97 is also implicated in the regulation of lysosomal and autophagosomal degradation (Papadopoulos and Meyer, 2017; Koerver et al., 2019; Ferrari et al., 2022).

Pathogenic VCP/p97 mutations cause a multisystem proteinopathy linked to different pathologies, such as inclusion bodies myopathy associated with Paget’s disease of the bone and FTD (IBMPFD) as well as ALS (Watts et al., 2004; Kimonis et al., 2008; Johnson et al., 2010; Gonzalez-Perez et al., 2012; Gang et al., 2016). VCP/p97 binds to protein aggregates and delivers misfolded proteins to the proteasome, which explains its crucial role in proteotoxic stress (van den Boom and Meyer, 2018). Notably, the peptide:N-glycanase and ubiquitin-associated (UBA)- or ubiquitin regulatory X (UBX) domain-containing proteins (PUB)-interacting motif (PIM) domain of VCP/p97 specifically interacts with the PUB domain of the M1-ubiquitin specific E3 ubiquitin ligase HOIP (Elliott et al., 2014; Schaeffer et al., 2014) and this interaction was shown to recruit HOIP and VCP/p97 to pathogenic protein aggregates in a feed-forward mechanism (van Well et al., 2019).

Several lines of evidence indicated a role for VCP/p97 in SG disassembly and clearance. Inactivation of Cdc48, the yeast ortholog of VCP/p97, resulted in the accumulation of heat stress-induced SGs due to defective autophagic clearance, an observation that was confirmed in several mammalian cellular models (Buchan et al., 2013). Moreover, Buchan et al. reported that the overexpression of pathogenic VCP/p97 mutants in HeLa cells induced the formation of constitutive SGs. Inhibition of VCP/p97 function has been reported to promote the colocalization of arsenite-induced SGs with ubiquitinated defective ribosomal products (DRiPs), which are released by disassembling polysomes and are usually degraded in a VCP/p97-dependent manner before SGs are formed (Seguin et al., 2014). This observation suggested that VCP/p97 also affects SG composition and morphology. Turakhiya et al. reported that the recruitment of VCP/p97 and the proteasome to arsenite-induced SGs is mediated by zinc finger AN1-type-containing protein 1 (ZFAND1) via its ubiquitin-like domain and zinc finger domain, respectively (Turakhiya et al., 2018). Deletion of ZFAND1 or inhibition of the proteasome caused the formation of aberrant and persistent SGs, characterized by the presence of DRiPs. These aberrant arsenite-induced SGs stained positive for p62 and microtubule-associated protein 1 light chain three beta (LC3B) and were cleared by autophagy (Turakhiya et al., 2018). Wang et al. (2019) observed that in response to heat stress and arsenite treatment, the autophagy-inducing kinases ULK1 and ULK2 localize to SGs and phosphorylate VCP/p97, thereby increasing its ATPase activity and promoting SG disassembly. Strikingly, disrupting ULK1/ULK2 expression in mice resulted in an inclusion body myopathy phenotype similar to that observed in mice expressing the pathogenic VCP/p97 mutant R155H or A232E (Weihl et al., 2007; Badadani et al., 2010; Custer et al., 2010; Wang et al., 2019). A critical role for VCP/p97 in heat stress-induced disassembly of SGs involving ubiquitination of G3BP1 was recently described by Gwon et al. (2021) see below. Also SUMOylation of VCP/p97 has been linked to SG dynamics. Inhibition of the proteasome or arsenite treatment induced SUMOylation of VCP/p97 in its NTD, which increased VCP/p97 hexamer assembly and its translocation to SGs and into the nucleus (Wang et al., 2016). These observations suggested that SUMOylation contributes to the fine-tuning of VCP/p97 functions under stress conditions.

### G3BPs

G3BPs are a family of RNA-binding proteins, which control mRNA stability and translation in response to environmental stress. This family includes three homologs in mammals, G3BP1, G3BP2a and its splice variant G3BP2b, which show a similar domain structure, comprising a nuclear transport factor 2 (NTF2)-like domain, followed by an acidic region (IDR1) and a proline-rich region (IDR2), an RNA recognition motif (RRM), and an arginine and glycine-rich region (RGG) at the C-terminus (IDR3). The NTF2-like domain mediates dimerization of G3BPs and protein-protein interactions, for example with the SG components Caprin-1 or USP10 that influence SG formation by positive (Caprin-1) or negative (USP10) cooperativity (Kedersha et al., 2016; Guillén-Boixet et al., 2020; Sanders et al., 2020; Yang et al., 2020). G3BPs show differences in the number of their proline-rich (PxxP) motifs. Whereas G3BP1 has only one PxxP motif, G3BP2a and G3BP2b have 4 and 5, respectively, which may influence the nature and extent of their interactions. G3BPs have been implicated in the regulation of various cellular processes, most notably in SG formation, antiviral responses, and cell viability and proliferation, linking G3BPs to disease mechanisms (Alam and Kennedy, 2019; Kang et al., 2021; Sidibé et al., 2021). For example, several viruses target G3BPs to evade innate immune responses of the host. Moreover, G3BPs expression levels are upregulated in some tumors, which has been correlated with cancer progression, invasiveness, and metastasis. Both G3BP1 and G3BP2 are core components of SGs that trigger SG formation in a context- and cell type-specific manner (Tourrière et al., 2003; Matsuki et al., 2013; Kedersha et al., 2016). A double knock-out (KO) of G3BP1 and G3BP2 in U2OS cells abolished SG formation in response to arsenite treatment but not in response to heat or osmotic stress (Yang et al., 2020). In fact, G3BP1 and G3BP2 seem to be the most important proteins in the SG interaction network that set the percolation threshold for LLPS (Guillén-Boixet et al., 2020; Sanders et al., 2020; Yang et al., 2020). Recent publications on the determinants of SG formation revealed that G3BPs function as scaffolds that sense local increases in the concentration of protein-free unfolded RNAs and form multivalent clusters on RNAs, resulting in a core protein-RNA interaction network (Guillén-Boixet et al., 2020; Yang et al., 2020). G3BPs are the central node of this network by acting as a molecular switch to trigger RNA-dependent LLPS under stress conditions. Under non-stress conditions, G3BP1 adopts an auto-inhibited closed conformation, characterized by an intramolecular electrostatic interaction between IDR1 and IDR3. When unfolded RNAs are released from polysomes in response to stress, G3BP1 undergoes a conformational transition into an open, multivalent conformation, allowing binding of RNAs to IDR3 (Guillén-Boixet et al., 2020; Yang et al., 2020). RNA-induced conformational switching of G3BP1 was shown to be inhibited by site-specific phosphorylation, most probably by favoring the auto-inhibited state (Guillén-Boixet et al., 2020; Sanders et al., 2020; Yang et al., 2020). Moreover, binding of RNAs to G3BP1 prevents RNA aggregation, suggesting a chaperoning function of G3BP1 to support RNA homeostasis (Guillén-Boixet et al., 2020).

PTMs of G3BPs can affect their valency and thus regulate SG dynamics, as has been shown for the phosphorylation of G3BP1 at S149 and S232 (Guillén-Boixet et al., 2020; Sanders et al., 2020; Tauber et al., 2020; Yang et al., 2020). Two recent studies discovered a role for G3BP1 ubiquitination in SG disassembly. Maxwell et al. (2021) performed a ubiquitinome analysis in mammalian cells in response to different stress conditions and found that ubiquitination is essential for the disassembly of SGs induced by heat stress but not by arsenite treatment. In this study, G3BP1 was detected as one of the SG components that is ubiquitinated in response to heat stress. Gwon et al. (2021) identified the critical lysine residues modified by ubiquitin and uncovered the mechanism of SG disassembly promoted by G3BP1 ubiquitination. By using ubiquitin mutants, linkage-specific ubiquitin antibodies, and G3BP1 lysine mutants, it was shown that heat stress mostly induced K63-linked polyubiquitination of six lysine residues localized in the NTF2-like domain. Expression of the G3BP1 lysine mutants in G3BP1/2 double KO cells or inhibition of ubiquitination by targeting the E1 enzyme UBA1 did not interfere with heat stress-induced assembly of SG but delayed their disassembly during recovery. Gwon et al. (2021) identified the ER-associated protein FAF2 as an interactor of ubiquitinated G3BP1 that recruits VCP/p97 to heat stress-induced SGs. VCP/p97 then extracts G3BP1 from SGs, thereby decreasing the percolation threshold and inducing SG disassembly.

## Conclusion

Tremendous progress has been made recently in unraveling the basic principles of SG assembly and disassembly by identifying the context-dependent proteome of SGs, characterizing interaction networks of SG-associated proteins and elucidating biophysical processes driving LLPS. However, we are only beginning to understand the role of specific PTMs in regulating these processes. PTMs may contribute to the contextual and possibly cell type-specific regulation of SG dynamics. Ubiquitination has emerged as a crucial determinant of SG disassembly after heat stress, promoting recovery from stress. Based on its role in mediating proteasomal and autophagosomal degradation, ubiquitination can also affect the clearance of SGs formed under persistent stress or due to pathogenic mutations of SG components. There is still some work ahead to uncover the role of specific ubiquitin linkages and heterotypic ubiquitin chains in these processes. Moreover, the identification of E3 ubiquitin ligases and deubiquitinases implicated in the regulation of SG dynamics is an important endeavor to exploit therapeutic strategies for diseases linked to aberrant SGs.
